# Supplementary material for: “Working Together”: Perspectives of Healthcare Professionals in Providing Virtual Care to Youth with Chronic Pain during the COVID-19 Pandemic
Source: Int J Environ Res Public Health. 2023 Mar 8;20(6):4757. doi: 10.3390/ijerph20064757 (PMC10048812; doi:10.3390/ijerph20064757)
Supplement: Supplementary file 1 [file ijerph-20-04757-s001.zip › ijerph-2225868-supplementary.pdf]

Section S1

Clinician Interview Guide

Instructions for the interviewer:

-Never cut off a participant. Always let the participant speak even if s/he is anticipating another question. In this case, it might be pertinent to ask that question earlier than suggested in the interview guide.

-If a participant's narrative becomes tangential, do not cut off the person, but rather reorient him/her toward an interview question when the opportunity presents itself (e.g., when they mention something that could be related to an interview question theme, or when there is a silence).

-Do not hesitate to follow-up on a question you have asked or to reformulate a question if someone believes they have nothing to say about a specific topic.

-Give enough time for the person to think and reflect so that they can answer the questions. Do not try to fill in the silences.

-Use terminology that participants are most familiar with. Avoid as much as possible scientific terminology, unless someone uses it spontaneously.

The goal of this interview is to understand your experiences as a clinician providing assessment and treatment through virtual care. I will ask you some questions so that you can tell me about your experience delivering virtual care.

The interview will be video recorded and kept private. Following the interview, I will review the transcript produced in TEAMS to check the accuracy and update it if needed. After the transcript is complete the video recording will be destroyed. If you would prefer to have me record without your camera on that is also an option. Additionally, you can stop the interview and withdraw from the study at any point. You may contact the Research Coordinator to request your interview data be removed from the study before analysis begins. Please let me know if a question makes you feel uncomfortable or if you would like to take a break. Alright, now I will start the video recording.

To start off, I would like to know about your role on the chronic pain team and how long you have been providing care virtually.

1. What is your role on the chronic pain care team?
2. How many months have you been providing care virtually? Was this your first experience providing virtual care?
3. How would you generally describe your experience providing virtual care?

Equipment and Technical Issues

In this next section, we would like to learn more about your experiences using video conferencing technology. Specifically, how comfortable you felt providing services over video conferencing and any technical challenges that came up.

4. How would you describe your overall experience using the videoconferencing software to deliver the virtual sessions?

Prompt/Follow-up

- Were you satisfied with the quality of the picture and sound during the sessions?
- Did you have concerns about using video conferencing technology?
- Did you experience any technical difficulties during your sessions?
- (If lots of technical difficulties) Did you feel that the technical challenges affected the quality of care you provided?

5. How did you generally feel about providing sessions over video conferencing?

Prompt/Follow-up

- Were there any benefits to discussing chronic pain issues over video?
- Did you have any concerns about privacy during sessions?
- Were there aspects of the virtual sessions that made treatment more difficult than if it were delivered in-person?

Communication and Rapport

Now, I am going to ask you about your experiences treating patients virtually.

6. Overall, how did you feel about meeting with patients virtually?

Prompt/Follow-up

- Were there any challenges in engaging with patients and their parents in this way?
- Were there issues with developing a therapeutic relationship with the patient and caregiver in the videoconference?
- Were there issues with patient focus and attention during virtual care sessions?
- Were there any challenges in addressing sensitive topics during virtual sessions?

Clinical Assessment

In this next section, I would like to hear about the assessment sessions you provided through virtual care.

7. Overall, did you feel that you were able to adequately assess your patients virtually?

Prompt/Follow-up

- Was there anything that made you doubt your ability to assess patients virtually?
- Were there any aspects of the assessment that you were not able to adequately complete in the virtual setting?
- Was the lack of physical contact with the patient an issue in your assessment?
- Were there aspects of virtual care that improved your assessment?
- Was there anything that you wish was different about the virtual assessment?

Clinical Treatment

Now, I would like to hear about the treatment sessions you provided through virtual care.

8. Overall, did you feel confident that you could provide treatment in the virtual session as effectively as if they were providing treatment in-person?

Prompt/Follow-up

- Were there issues with your ability to maintain patient and caregiver privacy during virtual treatment sessions?
- Was there anything that made you doubt your ability to treat your patients virtually?
- Was there anything that you wish was different about the virtual treatment sessions?

Final questions

In these final questions, I would like to know more about your overall evaluation of virtual care for chronic pain treatment.

9. How would you generally evaluate your experience providing virtual care as a whole?

Prompt/Follow-up

- Did virtual care change the way you were able to manage patients?
- Did virtual care change your ability to access patients?
- Is there anything you would recommend changing about the way the program was delivered?

10. Would your treatment preferences be different if we were not in a global pandemic?

Prompt/Follow-up

- Would you be more or less open to providing virtual care?

11. Is there anything else you would like to say?

Thank you very much for your time and your openness during this interview.

Section S2

HCP Satisfaction with Virtual Treatment Survey

Study Title: A New Paradigm: Intensive Functional Rehabilitation for Pediatric Pain through Virtual Care - Getting Children Back to Life During A Pandemic

Principal Investigator (Study Doctor):
Dr. Danielle Ruskin, PhD, C Psych, Department of Psychology and Department of Anesthesia and Pain Medicine, Hospital for Sick Children, (416) 813-8960 ext. 208960

Co-Investigator(s):
Jennifer Stinson, PhD, RN-EC, CPNP, Scientist, Child Health Evaluative Services, Hospital for Sick Children, (416) 813-7654 ext. 304514
Naiyi Sun, MD FRCP, Department of Anesthesia and Pain Medicine, Hospital for Sick Children, (416) 813-7445 ext. 207445
Lisa D'Alessandro, APN, Department of Anesthesia and Pain Medicine, Hospital for Sick Children, (416) 813-7654 ext. 202083
Giulia Mesaroli, PT, Department of Rehabilitation Services, Hospital for Sick Children, (416) 813-6755 ext. 206755
Catherine Munns, PhD,C Psych, Department of Psychology, Hospital for Sick Children, (416) 813-2370 ext. 202370
Monique Tremblay, MA, Department of Psychology, Hospital for Sick Children

Study Sponsor and/or Funder:
The study sponsor is the principal investigator, Dr. Danielle Ruskin. The study is funded by the Hospital for Sick Children, Pain Centre.

Conflict of Interest:
There are no conflicts of interest to declare related to this study.

You are being asked to participate because you are a healthcare provider in Canada who works with chronic pain patients to provide multidisciplinary pain treatment (MDT). This study examines the feasibility and effectiveness of Multidisciplinary (MDT) Pain treatment for chronic pain delivered through virtual care.

Your participation in this study will require the completion of the following questionnaire. This should take approximately 15 minutes of your time. The questionnaire asks questions about your satisfaction with providing MDT pain treatments virtually. Your participation will be anonymous and you will not be contacted again in the future. You will not be paid for being in this study. This survey involves minimal risk to you. The benefits, however, may impact society by helping increase knowledge about healthcare provider feedback on use of virtual care for chronic pain multi-disciplinary treatment.

All information collected about you will be de-identified by replacing your identifiable information (i.e., name) with a "study number". Only the "study code key" can connect the information collected about you to your identify. The study code key will be safeguarded by the SickKids research team. Even though the risk of identifying you from the study data is very small, it can never by completely eliminated.

You do not have to be in this study if you do not want to be. You do not have to answer any questions that you do not want to answer for any reason. If you have any questions about this project you may contact the study doctor Danielle Ruskin at (416) 813-8960 ext. 208960.

By completing this questionnaire, you are consenting to its use in research.

HCP Monthly Satisfaction Survey

Please complete the survey below.
Thank you!

Please answer the following questions based on your experience providing treatment sessions to parents and/or patients in virtual care.

**Survey completed by:**	○ Medicine<br>○ Nursing<br>○ Mental health services<br>○ Rehabilitation services<br>reset
**How many months have you been providing virtual care?**	Expand

Equipment and Technical Issues

Please answer the following questions based on your experience providing **treatment sessions** to parents and/or patients in virtual care.

**I was satisfied with the quality of the picture (video) during the videoconference.**	○ Strongly Disagree<br>○ Disagree<br>○ Somewhat Disagree<br>○ Neither Agree nor Disagree<br>○ Somewhat agree<br>○ Agree<br>○ Strongly Agree<br>reset
**I was satisfied with the quality of the sound (audio) during the videoconference.**	○ Strongly Disagree<br>○ Disagree<br>○ Somewhat Disagree<br>○ Neither Agree nor Disagree<br>○ Somewhat agree<br>○ Agree<br>○ Strongly Agree<br>reset
**I had easy access to videoconferencing technology.**	○ Strongly Disagree<br>○ Disagree<br>○ Somewhat Disagree<br>○ Neither Agree nor Disagree<br>○ Somewhat agree<br>○ Agree<br>○ Strongly Agree<br>reset
**I experienced few/no technical difficulties during the videoconference (e.g. unexpected disconnections, loss of sound or picture, etc.).**	○ Strongly Disagree<br>○ Disagree<br>○ Somewhat Disagree<br>○ Neither Agree nor Disagree<br>○ Somewhat agree<br>○ Agree<br>○ Strongly Agree<br>reset

Communication and Rapport

Please answer the following questions based on your experience providing **treatment sessions** to parents and/or patients in virtual care.

**I felt able to carry out effective treatment through the videoconferencing modality.**	◯ Strongly Disagree<br>◯ Disagree<br>◯ Somewhat Disagree<br>◯ Neither Agree nor Disagree<br>◯ Somewhat agree<br>◯ Agree<br>◯ Strongly Agree<br>reset
**I believe I was able to develop a therapeutic relationship with the patient and caregiver in the videoconference.**	◯ Strongly Disagree<br>◯ Disagree<br>◯ Somewhat Disagree<br>◯ Neither Agree nor Disagree<br>◯ Somewhat agree<br>◯ Agree<br>◯ Strongly Agree<br>reset
**Patients were as focused and attentive during virtual care sessions as they typically are during in-person care.**	◯ Strongly Disagree<br>◯ Disagree<br>◯ Somewhat Disagree<br>◯ Neither Agree nor Disagree<br>◯ Somewhat agree<br>◯ Agree<br>◯ Strongly Agree<br>reset
**It was no more challenging to discuss sensitive topics (e.g., self-injury) during virtual sessions (relative to in-person care).**	◯ Strongly Disagree<br>◯ Disagree<br>◯ Somewhat Disagree<br>◯ Neither Agree nor Disagree<br>◯ Somewhat agree<br>◯ Agree<br>◯ Strongly Agree<br>reset

Clinical Assessment	
**I was confident that I could assess the patient as effectively through video as I would be if I was there in-person.**	○ Strongly Disagree<br>○ Disagree<br>○ Somewhat Disagree<br>○ Neither Agree nor Disagree<br>○ Somewhat agree<br>○ Agree<br>○ Strongly Agree<br>reset
**I believe I was able to gain sufficient subjective and objective information of the patient's presenting issues (physical or mental health) in the virtual assessment to make a diagnosis.**	○ Strongly Disagree<br>○ Disagree<br>○ Somewhat Disagree<br>○ Neither Agree nor Disagree<br>○ Somewhat agree<br>○ Agree<br>○ Strongly Agree<br>reset
**I was able to maintain patient and caregiver privacy during the virtual clinic assessment.**	○ Strongly Disagree<br>○ Disagree<br>○ Somewhat Disagree<br>○ Neither Agree nor Disagree<br>○ Somewhat agree<br>○ Agree<br>○ Strongly Agree<br>reset
**The lack of physical contact in a virtual clinic is NOT a problem for managing chronic pain.**	○ Strongly Disagree<br>○ Disagree<br>○ Somewhat Disagree<br>○ Neither Agree nor Disagree<br>○ Somewhat agree<br>○ Agree<br>○ Strongly Agree<br>reset
**Were there aspects of the assessment that you feel you missed out on due to virtual delivery (e.g., behavioural cues)?**	○ Yes<br>○ No<br>reset
**Please specify the aspects of the assessment that you feel you missed out on:**	Expand
**Were there aspects of virtual care that aided your assessment (e.g., the opportunity to see aspects of the home environment that may be contributing to/maintaining client's pain symptoms)?**	○ Yes<br>○ No<br>reset
**Please specify the aspects of the virtual care that aided your assessment:**	

Program Evaluation	
**Virtual care improves my access to patients managing chronic pain.**	○ Strongly Disagree<br>○ Disagree<br>○ Somewhat Disagree<br>○ Neither Agree nor Disagree<br>○ Somewhat agree<br>○ Agree<br>○ Strongly Agree<br>reset
**Virtual care improves the management of patients who have chronic pain.**	○ Strongly Disagree<br>○ Disagree<br>○ Somewhat Disagree<br>○ Neither Agree nor Disagree<br>○ Somewhat agree<br>○ Agree<br>○ Strongly Agree<br>reset
**I am confident that I can provide effective treatment for my patient's needs over virtual care.**	○ Strongly Disagree<br>○ Disagree<br>○ Somewhat Disagree<br>○ Neither Agree nor Disagree<br>○ Somewhat agree<br>○ Agree<br>○ Strongly Agree<br>reset
**Which of the following best describes your preferences for virtual care:**	○ I would prefer to have all sessions delivered via virtual care again in the future even if I had the opportunity to schedule an in-person appointment.<br>○ I would prefer to have an in-person assessment, but I would be open to having the remainder of the sessions delivered via virtual care.<br>○ I would prefer to have a hybrid model of in-person and virtual care delivery (e.g., 2-3 sessions delivered in-person and the remainder delivered virtually).<br>○ I would prefer to have all sessions delivered in-person in the future.<br>reset
**I believe that virtual care has improved patient attendance at sessions (i.e., fewer missed appointments, greater access to parents in treatment, etc.).**	○ Strongly Disagree<br>○ Disagree<br>○ Somewhat Disagree<br>○ Neither Agree nor Disagree<br>○ Somewhat agree<br>○ Agree<br>○ Strongly Agree<br>reset
**Do you think you will continue using virtual care?**	○ Yes, but only minimally.<br>○ Yes, but only 50% of time.<br>○ Yes, more than 80% of the time.<br>○ No, not at all.<br>reset

**Virtual care has provided new opportunities for treatment (e.g., shaping behaviours within the home environment, improved use of technology in sessions).**	○ Strongly Disagree<br>○ Disagree<br>○ Somewhat Disagree<br>○ Neither Agree nor Disagree<br>○ Somewhat agree<br>○ Agree<br>○ Strongly Agree<br>reset
**The ability to provide treatment over virtual care has had personal benefits (e.g., ability to work from home, better time management).**	○ Strongly Disagree<br>○ Disagree<br>○ Somewhat Disagree<br>○ Neither Agree nor Disagree<br>○ Somewhat agree<br>○ Agree<br>○ Strongly Agree<br>reset
**If you have any comments or suggestions about virtual care, please note here.**	Expand
